# Supplementary material for: Migrating mule deer compensate en route for phenological mismatches
Source: Nat Commun. 2023 Apr 10;14:2008. doi: 10.1038/s41467-023-37750-z (PMC10086060; doi:10.1038/s41467-023-37750-z)
Supplement: Supplementary file 1 — Supplementary Information [file 41467_2023_37750_MOESM1_ESM.pdf]

Supplementary Information

Supplementary Methods

Delineation of migratory routes and seasonal ranges

We were unable to collect complete GPS data from n = 24 animal-years with store-on-board GPS collars either because of collar failures or because we did not recapture the deer and were unable to download GPS data during captures. We removed these animal-years in addition to n = 36 animal-years with incomplete spring migrations because of collar failures or mortalities before or during spring migration. We also removed n = 5 animal-years that spent the winter approximately 70 km north from their historic winter ranges in the Red Desert, n = 10 animal-years that were captured for the first time on their migratory route without prior GPS data, n = 4 animal-years that were recaptured during migration, n = 2 animal-years that returned extremely late to their winter ranges in the Red Desert on 17 March and 26 March, and n = 1 outlier that migrated >300 km to a summer range in eastern Idaho. We only documented the complete spring migration of n = 1 deer in 2013 and n = 1 deer in 2015. We therefore removed these two deer from our analyses.

Factors influencing the start of spring migration

Because our first GLMM only included n = 50 mule deer (n = 93 animal-years) with known age, nutritional condition, and pregnancy, we conducted a second GLMM to verify that migration distance did not influence start of spring migration across all n = 152 animal-years within our movement analyses. Based on our second GLMM, migration distance did not influence the start of spring migration (β = 0.10, 95% CI = -0.08–0.27).

Cue-responses on winter range

We used a mixed effects Cox Proportional Hazards (CPH) model to evaluate the effect of changes in plant phenology, temperature, snow depth, and photoperiod on the potential that deer would initiate spring migration. We removed *n* = 31 animal-years from the CPH model because they began spring migration before 20 March (standardized start time). NDVI was strongly correlated with photoperiod ($R^2 = 0.7$). We removed photoperiod and retained NDVI in the CPH model because we were interested in quantifying the synchronicity between movement of deer and plant phenology. The VIFs for all other covariates in the CPH model (i.e., NDVI, temperature, snow depth, daily change in NDVI, daily change in temperature, daily change in snow depth) were similar and relatively small (< 2.0). The baseline hazard varied across time relative to NDVI, temperature, and snow depth, which violated the key assumption of a CPH model that the baseline hazard remains proportional for each covariate[1,2]. To address these violations, we added three interaction terms between time and NDVI, temperature, and snow depth[1,2]. Fixed effects in the final CPH model (AIC = 887.81) included NDVI, temperature, snow depth, daily changes in NDVI, NDVI*time, and snow depth*time. Daily changes in temperature, daily changes in snow depth, and temperature*time did not contribute significantly to the CPH model.

Supplementary Discussion

Cue-responses on winter range

Mule deer were more likely to depart their winter ranges with decreases in NDVI and increases in temperature. Although NDVI and temperature influenced the instantaneous potential of spring migration for mule deer, individual cue-responses to NDVI ($7.69 \times 10^{-19} \pm 7.56 \times 10^{-5}$ [$\bar{x} \pm$ 95%

CI]) and temperature ($1.41 \times 10^{-19} \pm 5.53 \times 10^{-6}$) were relatively small, which indicated weak cue responses at the individual level. The coefficient for NDVI was negative, and thus, can be interpreted as either (1) deer were more likely to start migrating with decreases in NDVI, or (2) deer were less likely to start migrating with increases in NDVI. Nonetheless, out of the $n = 121$ animal-years in our CPH model, only $n = 4$ animal-years started spring migration 11 ± 2 days behind peak NDVI while $n = 117$ animal-years started spring migration 38 ± 4 days ahead peak NDVI. Because the vast majority of animals (97%) departed winter range ahead peak NDVI, we interpreted the NDVI hazards ratio as deer being more likely to depart winter ranges when NDVI was low and not because NDVI had already reached its peak and was declining.

Previous work on this population of mule deer found a negative effect of migration distance on the start of spring migration across all migratory tactics (i.e., short-distance migrants [0–50km], medium-distance migrants [50–150 km], long-distance migrants [150–250 km][3]). We evaluated the behavioral ability of long-distance migrants (herein, classified as deer that migrated >130 km) to compensate for phenological mismatches with the green wave during migration. Our results indicate that migration distance does not influence start of spring migration for long-distance migrants in this population.

The precise factors that influence variation in the start of spring migration for mule deer wintering in the Red Desert remain unclear. Other variables, such as population density and social cues, could determine when animals depart their winter range[4,5]. Each spring, an estimated 500–1,000 mule deer migrate from their winter ranges in the Red Desert to their summer ranges in northwestern Wyoming[6]. During migration, these deer join an additional 4,000–5,000 migrating mule deer that spend the winter along the foothills of the Wind River Range[6]. A mass migration in which all mule deer leave their winter ranges at the same time may be not be

feasible nor beneficial within this narrow migration corridor. Regardless of the underlying mechanisms that influence the start of spring migration, mule deer departed winter ranges at different times relative to peak green-up and experienced phenological mismatches with the green wave at the onset of spring migration.

Supplementary Figures

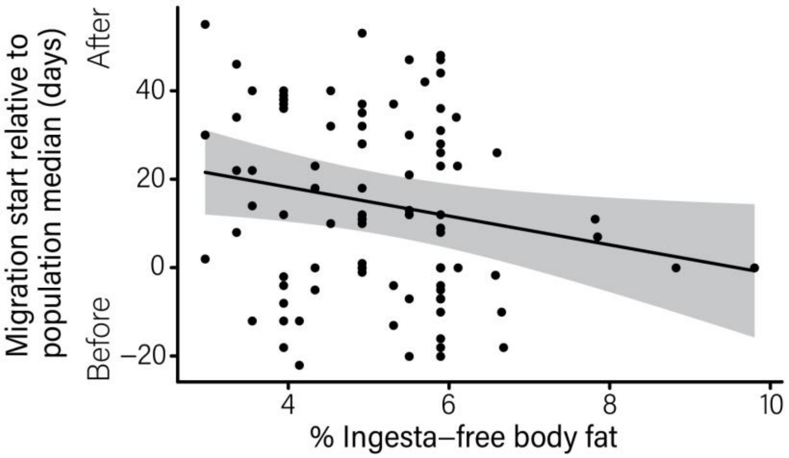

Supplementary Fig. 1. Effect of percent ingesta-free body fat in March on the start of spring migration. Based on a generalized linear mixed model (GLMM), percent ingesta-free body fat negatively influenced the start of spring migration (predicted coefficients ± 95% confidence interval), although the relationship was relatively weak (β = -3.25, 95% CI = -6.28–(-0.34)). Based on a one-way analysis of variance (ANOVA), percent ingesta-free body fat did not differ among early, mid, and late migrants ($IFBFat_{early}$ = 5.41 ± 0.45 %; $IFBFat_{mid}$ = 5.04 ± 0.28 %; $IFBFat_{late}$ = 5.00 ± 0.41 %; $F_{2,112}$ = 1.32, p = 0.27).

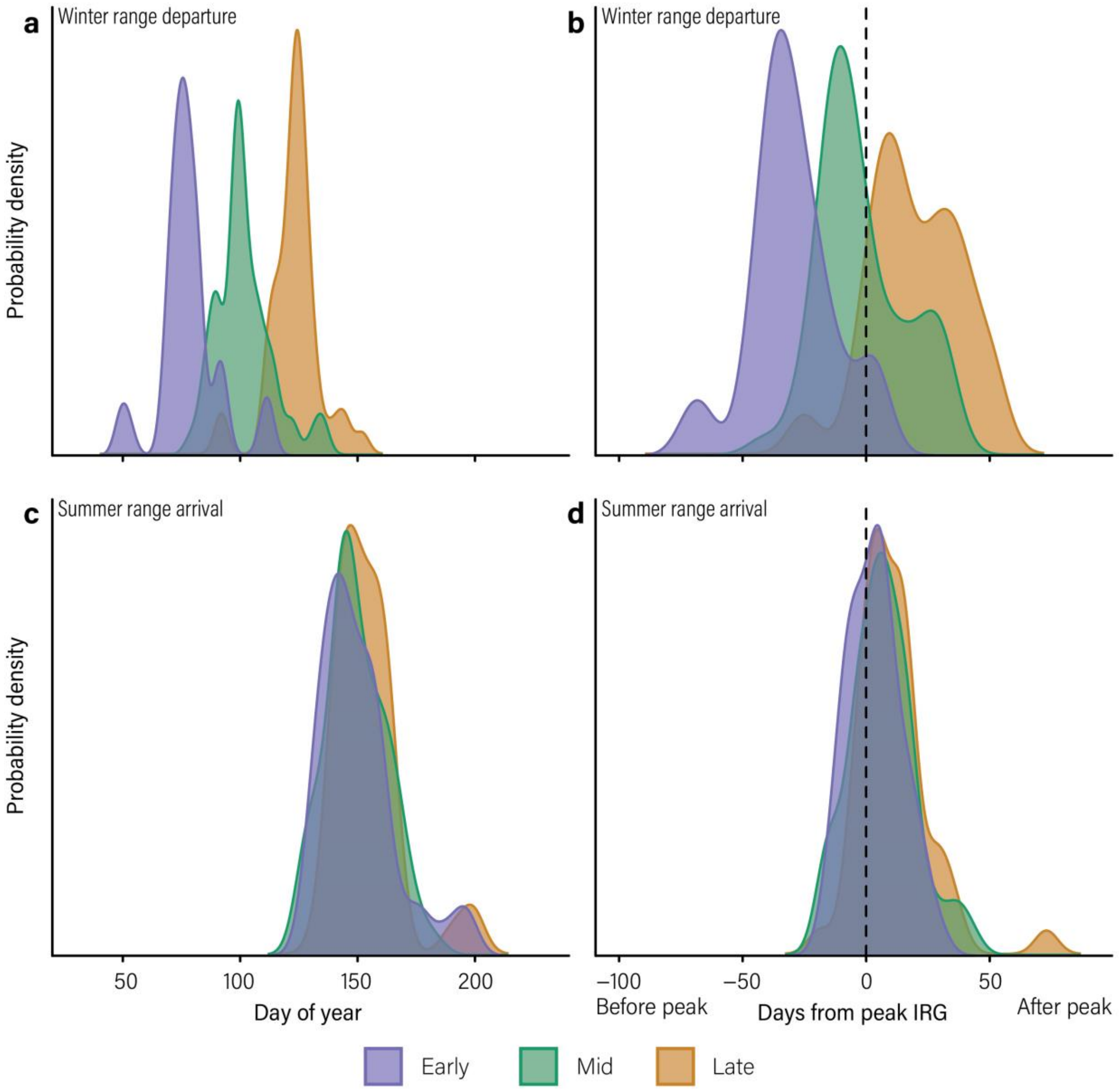

Supplementary Fig. 2. Comparisons in the location of early, mid, and late migrants along the green wave at the start and end of spring migration. a Start date of spring migration among early migrants (*n* = 47 animal-years; purple), mid-migrants (*n* = 58 animal-years; green), and late migrants (*n* = 47 animal-years; orange). Early, mid, and late migrants varied in the timing of migration and how far they were from peak Instantaneous Rate of Green-up (IRG) at the start of spring migration (MANOVA, $F_{2,149}$ = 20.54, Pillai's Trace = 0.72, p = 1.19 x 10^{-24}).

On average, early migrants departed their winter ranges on 19 March ± 4 days ($\bar{x}$ ± 95% CI), mid-migrants departed their winter ranges on 12 April ± 3 days, and late migrants departed their winter ranges on 3 May ± 3 days. **b** Days from peak IRG (vertical black line) at the start of spring migration. Early and late migrants were mismatched with the green wave at the onset of migration. **c** End date of spring migration among early, mid, and late migrants. On average, early migrants arrived at their summer ranges on 30 May ± 5 days, mid-migrants arrived at their summer ranges on 29 May ± 3 days, and late migrants arrived at their summer ranges on 3 June ± 4 days. **d** Days from peak IRG at the end of spring migration. Early and late migrants compensated for being mismatched with green wave because they ended their spring migration closer to peak IRG than when they started spring migration.

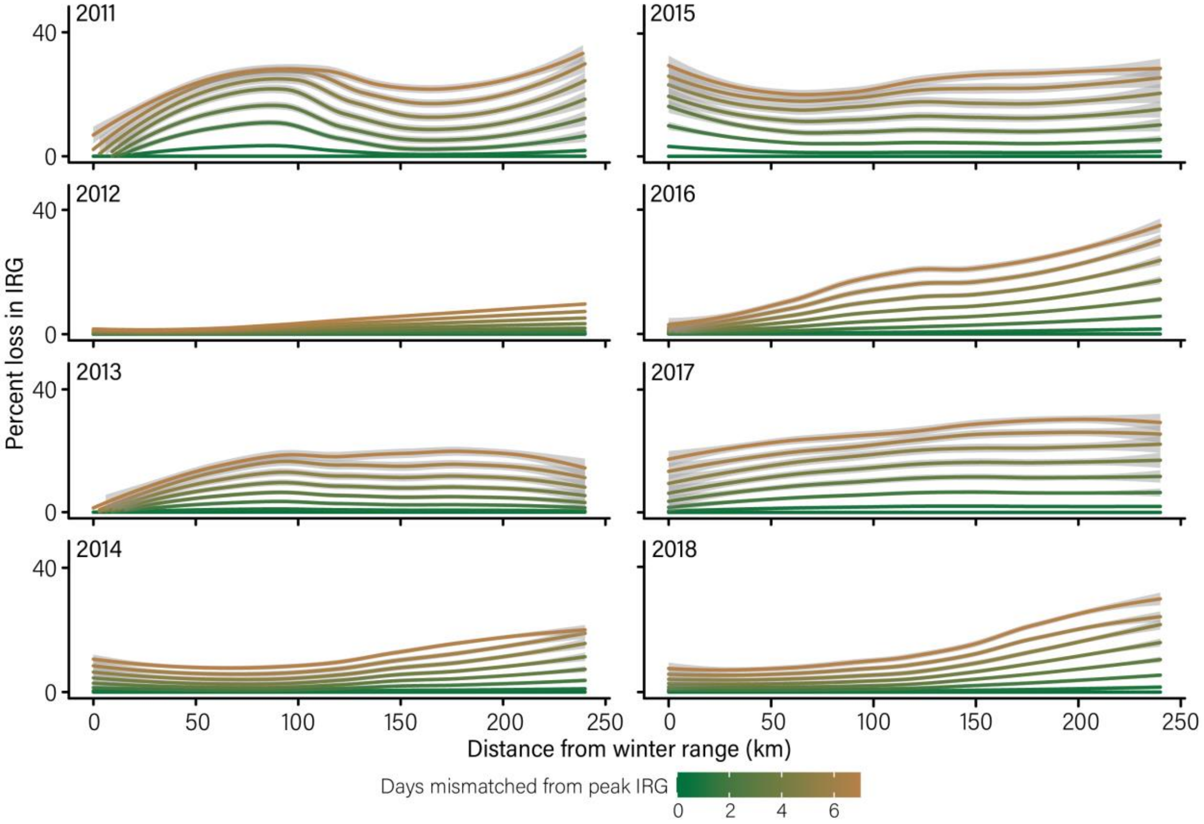

Supplementary Fig. 3. Loss in Instantaneous Rate of Green-up (IRG) as a function of distance from winter range (km) and days from peak IRG from 2011–2020. Loss in IRG depended on the degree of mismatch (i.e., days from peak IRG) and distance along the migration corridor (GAMM, $p < 2.20 \times 10^{-16}$). The foraging pentality of being mismatched by 7 days is 11% higher in the last quarter of migration than the first quarter of migration. Comparatively, the foraging penality of being mismatched from the green wave by 1 day is 0.6% higher in the last quarter of migration than the first quarter of migration. Losses in IRG were estimated for each pixel within the designated 240-km migration corridor of the Sublette Mule Deer Herd, western Wyoming, USA. Mean losses in IRG were plotted using a loess regression (grey bands represent 95% confidence intervals).

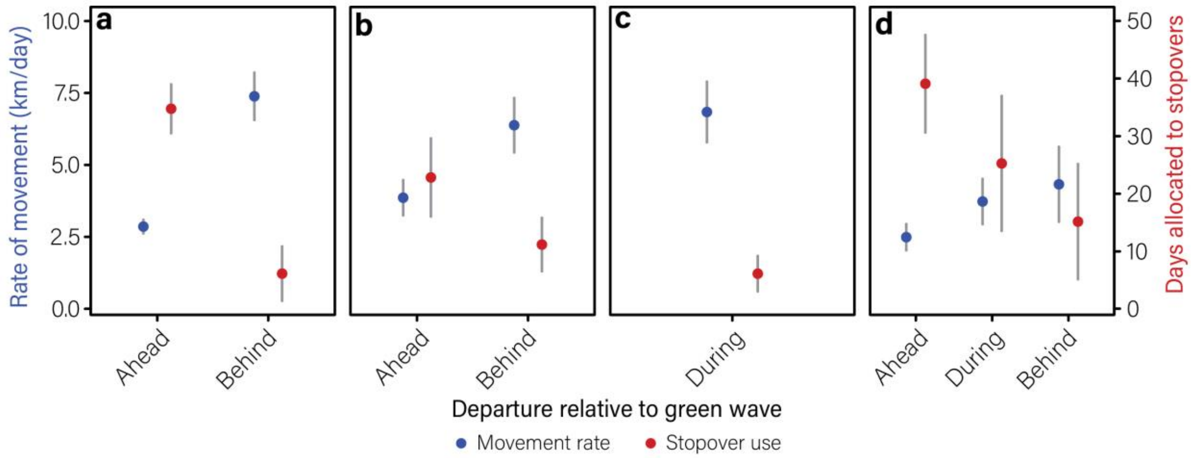

Supplementary Fig. 4. Comparisons of mean movement rate (km/day; blue) and mean stopover use (total days; red) ± 95 confidence intervals among mule deer that departed winter range ahead, during, or behind peak Instantaneous Rate of Green-up (IRG). Mule deer were classified as **a** full compensators (*n* = 78 animal-years), **b** partial compensators (*n* = 35 animal-years), **c** perfect surfers (*n* = 16 animal-years), and **d** non-compensators (*n* = 23 animal-years). Mule deer that started spring migration ahead peak IRG fully or partially compensated for phenological mismatches with the green wave by decelerating their movement and spending more time on stopovers. Comparatively, mule deer that started spring migration behind peak IRG fully or partially compensated for phenological mismatches with the green wave by accelerating their movement and spending less time on stopovers. Movement rate and stopover use varied among full compensators, partial compensators, perfect surfers, and non-compensators that departed winter range ahead, during, or behind peak IRG ($ANOVA_{rate}$, $F_{7,144} = 29.85$, $p = 3.58 \times 10^{-25}$; $ANOVA_{stopover}$, $F_{7,119} = 14.85$, $p = 8.24 \times 10^{-14}$). Full compensators that departed winter range behind peak IRG moved at a faster rate (7.38 ± 0.82 km/day) and allocated less time to stopovers (6 ± 5 days) than full compensators that departed winter range ahead peak IRG (2.86 ± 0.24 km/day; stopover use: 35 ± 4 days; Tukey's HSD_{rate}, $p < 2.20 \times 10^{-16}$; Tukey's $HSD_{stopover}$, $p = 2.29 \times 10^{-10}$).

Supplementary Tables

Supplementary Table 1. Mean days from peak Instantaneous Rate of Green-up (Days-From-Peak) ± 95% confidence intervals at the start and end of spring migration for *n* = 72 adult female mule deer (*n* = 152 animal-years) in western Wyoming, USA, 2011–2020.

		Start of spring migration		End of spring migration	
Year	*n*	Days-From-Peak	95% CI	Days-From-Peak	95% CI
2011	6	-21.39	8.78	7.31	10.85
2012	6	-11.02	16.52	5.16	7.09
2014	5	6.77	4.65	2.48	6.86
2016	12	11.24	13.92	22.59	12.12
2017	36	17.13	7.89	5.54	4.26
2018	37	-5.45	6.81	10.28	2.70
2019	26	-20.25	10.57	5.30	4.43
2020	24	-17.73	7.18	-0.52	4.46

Supplementary Table 2. Model output from a series of two-sided z-tests to determine whether *n* = 72 adult female mule deer (*n* = 152 animal-years) at the population level started and ended spring migration ahead or behind peak Instantaneous Rate of Green-up (IRG).

		Start of spring migration		End of spring migration	
Year	*n*	Z-value	P-value	Z-value	P-value
2011	6	-4.78	1.78×10^{-6}	1.32	0.19
2012	6	-1.31	0.19	1.43	0.15
2014	5	2.85	0.004	0.71	0.48
2016	12	1.58	0.11	3.65	0.0003
2017	36	4.25	2.09×10^{-5}	2.55	0.01
2018	37	-1.57	0.12	7.46	8.89×10^{-14}
2019	26	-3.76	0.0002	2.34	0.02
2020	24	-4.84	1.30×10^{-6}	-0.23	0.82

Supplementary Table 3. Output from the drop1 function for the full generalized linear mixed model (GLMM) that evaluated the effect of migration distance (km), age, nutritional condition (% scaled ingesta-free body fat [IFBFat] in March), and pregnancy (i.e., fetal rate [number of

fetuses per deer], fetal eye diameter [mm]) on the start of spring migration for *n* = 50 adult female mule deer (*n* = 93 animal-years) in western Wyoming, USA, 2011–2020.

Dropped covariate	AIC	LRT	Pr(Chi)
None	815.11		
Migration distance (km)	813.11	1.60×10^{-2}	0.97
Age	813.14	0.03	0.87
IFBFat (%)	818.19	5.08	0.02
Fetal rate (# fetuses per deer)	813.30	0.19	0.66
Fetal eye diameter (mm)	815.23	2.12	0.15

Note: AIC is the Akaike Information Criterion and LRT is the likelihood ratio test.

Supplementary Table 4. Model output from a mixed effects Cox Proportional Hazards (CPH) model used to identify environmental variables that trigger the start of spring migration. The final CPH model contained *n* = 3,645 daily observations and *n* = 121 events for *n* = 69 adult female mule deer (>1-yr-old) in western Wyoming, USA, 2011–2020.

Covariate	Coefficient estimate	Hazard ratio	SE	Z-value	P-value
NDVI	-4.79	0.01	1.44	-3.32	0.0009
Temperature (°C)	0.07	1.07	0.03	1.99	0.05
Snow depth (m)	-3.94	0.02	5.42	-0.73	0.47
Daily change in NDVI	-2.38	0.09	1.27	-1.88	0.06
NDVI: time	0.11	1.12	0.05	2.36	0.02
Snow depth: time	-0.22	0.80	0.27	-0.82	0.41

Supplementary Table 5. The rate of change in date of peak Instantaneous Rate of Green-up (IRG) and model outputs from linear regressions between mean date of peak IRG and distance along the 240-km migration corridor from 2011–2020. In 5 of the 8 tracking years, the rate of change in mean date of peak IRG was negative for the first 32 km migration, indicating a non-consecutive green wave that propagated backwards towards winter range.

Year	Distance along migration corridor (km)	Rate of change in date of peak IRG	Linear regression between date of peak IRG and distance
2011	0–30	Negative	$F_{1,29} = 2.86$, $R^2 = 0.06$, $p = 0.10$

	31–240	Positive	$F_{1,208} = 1057$, $R^2 = 0.83$, $p = 1.70 \times 10^{-83}$
2012	0–61	Positive	$F_{1,60} = 64.95$, $R^2 = 0.51$, $p = 3.88 \times 10^{-11}$
	62–106	Negative	$F_{1,43} = 104.1$, $R^2 = 0.70$, $p = 4.68 \times 10^{-13}$
	107–240	Positive	$F_{1,132} = 193$, $R^2 = 0.59$, $p = 1.35 \times 10^{-27}$
2014	0–20	Negative	$F_{1,19} = 0.06$, $R^2 = -0.05$, $p = 0.81$
	21–240	Positive	$F_{1,218} = 1179$, $R^2 = 0.84$, $p = 6.67 \times 10^{-90}$
2016	0–32	Negative	$F_{1,31} = 0.01$,$R^2 = -0.03$, $p = 0.92$
	33–240	Positive	$F_{1,206} = 522.8$, $R^2 = 0.72$, $p = 1.97 \times 10^{-58}$
2017	0–240	Positive	$F_{1,239} = 1949$, $R^2 = 0.89$, $p = 6.65 \times 10^{-117}$
2018	0–25	Negative	$F_{1,24} = 34.06$, $R^2 = 0.57$, $p = 5.11 \times 10^{-6}$
	26–240	Positive	$F_{1,213} = 1119$,$R^2 = 0.84$, $p = 9.89 \times 10^{-87}$
2019	0–240	Positive	$F_{1,239} = 1388$, $R^2 = 0.85$, $p = 1.65 \times 10^{-101}$
2020	0–21	Negative	$F_{1,20} = 3.38$, $R^2 = 0.10$, $p = 0.08$
	22–240	Positive	$F_{1,217} = 1118$, $R^2 = 0.84$, $p = 1.47 \times 10^{-87}$

Supplementary Table 6. The mean, min, max, 25% quartile and 75% quartile start of spring migration for *n* = 72 adult female mule deer (*n* = 152 animal-years) that migrated 134–293 km from their winter ranges in the Red Desert of south-central Wyoming to their summer ranges in northwestern Wyoming, USA. Early migrants started spring migration within the 25% quartile of start dates. Mid-migrants migrants started spring migration between the 25% and 75% quartiles of start dates. Late migrants started spring within the 75% quartile of start dates.

Year	*n*	Start of spring migration (day of year)	SE	95% CI	Min	Max	25% quartile	75% quartile
2011	6	97	5	11	90	124	91	92
2012	6	94	8	15	70	115	78	109
2014	5	115	2	5	110	122	112	119
2016	12	116	7	14	75	152	96	136
2017	36	105	3	7	74	134	86	123
2018	37	97	3	6	68	130	82	113
2019	26	97	5	10	49	145	78	120
2020	24	98	3	7	71	126	82	111

Supplementary Table 7. Model outputs from a series of Tukey's honestly significant different (HSD) tests, which were used as *post hoc* analyses from a one-way multivariate analysis of variance (MANOVA) to evaluate pairwise differences in the start date and end date of spring

migration, along with pairwise differences in days from peak Instantaneous Rate of Green-Up (Days-From-Peak), among adult female mule deer (>1-yr-old) that were early migrants (*n* = 47 animal-years), mid-migrants (*n* = 58 animal-years), or late migrants (*n* = 47 animal-years).

Response variable	Pairwise comparison	Pairwise difference among means	95% CI	P-value
Start date	late-early	44.83	39.08–50.58	$<2.20 \times 10^{-16}$
	mid-early	23.38	17.92–28.85	$<2.20 \times 10^{-16}$
	mid-late	-21.45	-26.91–(-15.98)	$<2.20 \times 10^{-16}$
End date	late-early	3.85	-3.02–10.73	0.38
	mid-early	-0.87	-7.41–5.67	0.95
	mid-late	-4.72	-11.26–1.82	0.21
Days-From-Peak at start	late-early	49.71	41.06–58.37	$<2.20 \times 10^{-16}$
	mid-early	28.27	20.03–36.50	4.61×10^{-13}
	mid-late	-21.45	-29.68–(-13.22)	1.86×10^{-8}
Days-From-Peak at end	late-early	7.40	1.09–13.72	0.02
	mid-early	3.12	-2.89–9.13	0.44
	mid-late	-4.28	-10.29–1.72	0.21

Supplementary Table 8. Mean date of peak Instantaneous Rate of Green-up (IRG) on all winter and summer ranges for *n* = 72 adult female deer (*n* = 152 animal-years) in western Wyoming, USA from 2011–2020. Annual deviation from mean date of peak IRG was calculated as the difference between date of peak IRG within a given year and mean date of peak IRG throughout the duration of the study on winter range (17 April) or summer range (24 May). Negative values indicate an early green-up of plants compared with the long-term average; whereas, positive values indicate a late green-up of plants.

Seasonal range	Year	Date of peak IRG	Deviation from mean date of peak IRG (days)
Winter range	2011	28 April	11
	2012	15 April	-2
	2014	18 April	1
	2016	15 April	-2
	2017	29 March	-19
	2018	12 April	-5

	2019	28 April	11
	2020	25 April	8
Summer range	2011	6 June	13
	2012	11 May	-13
	2014	22 May	-2
	2016	15 May	-9
	2017	27 May	3
	2018	16 May	-8
	2019	1 June	8
	2020	30 May	6

Note: Annual variation in date of peak IRG on winter range did not influence how far deer were from peak green-up at the start and end of spring migration ($\sigma_{winterIRG}$: $F_{1,150} = 0.25$, $R^2 = -0.005$, $p = 0.62$; $\sigma_{summerIRG}$: $F_{1,150} = 0.63$, $R^2 = -0.002$, $p = 0.43$), suggesting that deer arrived to summer range close to peak IRG irrespective of whether green-up was early or late.

Supplementary Table 9. Output from a generalized linear mixed model (GLMM) that evaluated the effect of migration distance (km) and intrinsic variables, including age, nutritional condition (% scaled ingesta-free body fat [IFBFat] in March), and pregnancy (i.e., fetal rate [number of fetuses per deer], fetal eye diameter [mm]) on overall movement rate (km/day) for *n* = 50 adult female mule deer (*n* = 93 animal-years) in western Wyoming, USA, 2011–2020.

Covariate	Coefficient estimate	SE	T-value	95% CI
Migration distance	0.01	0.01	0.76	-0.02–0.03
Age	-0.11	0.10	-1.13	-0.31–0.08
% IFBFat in March	-0.09	0.17	-0.52	-0.41–0.24
Fetal rate	-0.18	0.46	-0.39	-1.07–0.70
Fetal eye diameter	-0.01	0.14	0.07	-0.27–0.28

Supplementary Table 10. Output from a generalized linear mixed model (GLMM) that evaluated the effect of migration distance (km) and intrinsic variables, including age, nutritional condition (% scaled ingesta-free body fat [IFBFat] in March), and pregnancy (i.e., fetal rate [number of fetuses per deer], fetal eye diameter [mm]) on stopover use (total number of days allocated to high-use stopovers [≥3 days]) for *n* = 48 adult female mule deer (*n* = 85 animal-years) in western Wyoming, USA, 2011–2020.

Covariate	Coefficient estimate	SE	T-value	95% CI
Migration distance	-0.03	0.08	-0.38	-0.18–0.13
Age	0.40	0.67	0.61	-0.87–1.69
% IFBFat in March	0.70	1.23	-0.57	-3.05–1.65
Fetal rate	-1.74	3.23	-0.54	-7.91–4.55
Fetal eye diameter	-1.43	1.01	-1.42	-3.36–0.57

Supplementary Table 11. Sample size (*n*) and mean days from peak Instantaneous Rate of Green-up (Days-From-Peak) ± 95% confidence intervals at the start and end of spring migration for adult female mule deer (>1-yr-old) that were classified as full compensators, partial compensators, perfect surfers, or non-compensators and started spring migration ahead, behind, or during peak green-up (i.e., ± 7 days from peak green-up).

Classification of behavioral compensation	Start of spring migration relative to peak green-up	*n*	Days-From-Peak at start of spring migration (± 95% CI)	Days-From-Peak at end of spring migration (± 95% CI)
Full compensators	Ahead	50	32.39 ± 2.71	8.60 ± 1.54
	Behind	28		
Partial compensators	Ahead	16	14.19 ± 2.11	12.94 ± 2.48
	Behind	19		
Perfect surfers		16	3.66 ± 0.92	3.56 ± 0.94
Non-compensators	Ahead	5	10.79 ± 4.51	22.16 ± 6.07
	During	13		
	Behind	5		

Supplementary References

1. Fox, J. "Cox for proportional-hazards regression for survival data (Appendix)" in *An R and S-Plus companion to applied regression* (Sage Publications, Thousand Oaks, CA, 2002).
2. Rivrud, I. M. et al. Leave before it's too late: anthropogenic and environmental triggers of autumn migration in a hunted ungulate population. *Ecology* **97**, 1058–1068 (2016).
3. Sawyer, H., Middleton, A. D., Hayes, M. M., Kauffman, M. J. & Monteith, K. L. The extra mile: ungulate migration distance alters the use of seasonal range and exposure to anthropogenic risk. *Ecosphere* **7**, https://doi.org/10.1002/ecs2.1534 (2016).
4. Mysterud, A. et al. Partial migration in expanding red deer populations at northern latitudes – a role for density dependence? *Oikos* **120**, 1817–1825 (2011).
5. Berg, J. E., Hebblewhite, M., St. Clair, C. C. & Merrill, E. H. Prevalence and mechanisms of partial migration in ungulates. *Frontiers in Ecology and Evolution* **7**, https://doi.org/10.3389/fevo.2019.00325 (2019).

6. Kauffman, M. J. et al. *Wild Migrations: Atlas of Wyoming’s Ungulates* (Oregon State University Press, Corvallis, OR, 2018).
