# Supplementary material for: Migrating mule deer compensate en route for phenological mismatches
Source: Nat Commun. 2023 Apr 10;14:2008. doi: 10.1038/s41467-023-37750-z (PMC10086060; doi:10.1038/s41467-023-37750-z)
Supplement: Supplementary file 3 — Reporting Summary [file 41467_2023_37750_MOESM3_ESM.pdf]

nature portfolio

Corresponding author(s): Anna C. Ortega
Last updated by author(s): 03-21-2023

Reporting Summary

Nature Portfolio wishes to improve the reproducibility of the work that we publish. This form provides structure for consistency and transparency in reporting. For further information on Nature Portfolio policies, see our Editorial Policies and the Editorial Policy Checklist.

Statistics

For all statistical analyses, confirm that the following items are present in the figure legend, table legend, main text, or Methods section.

n/a	Confirmed	
☐	☒	The exact sample size (n) for each experimental group/condition, given as a discrete number and unit of measurement
☐	☒	A statement on whether measurements were taken from distinct samples or whether the same sample was measured repeatedly
☐	☒	The statistical test(s) used AND whether they are one- or two-sided<br>*Only common tests should be described solely by name; describe more complex techniques in the Methods section.*
☐	☒	A description of all covariates tested
☐	☒	A description of any assumptions or corrections, such as tests of normality and adjustment for multiple comparisons
☐	☒	A full description of the statistical parameters including central tendency (e.g. means) or other basic estimates (e.g. regression coefficient) AND variation (e.g. standard deviation) or associated estimates of uncertainty (e.g. confidence intervals)
☐	☒	For null hypothesis testing, the test statistic (e.g. F, t, r) with confidence intervals, effect sizes, degrees of freedom and P value noted<br>*Give P values as exact values whenever suitable.*
☒	☐	For Bayesian analysis, information on the choice of priors and Markov chain Monte Carlo settings
☒	☐	For hierarchical and complex designs, identification of the appropriate level for tests and full reporting of outcomes
☒	☐	Estimates of effect sizes (e.g. Cohen's d, Pearson's r), indicating how they were calculated

Our web collection on statistics for biologists contains articles on many of the points above.

Software and code

Policy information about availability of computer code

Data collection	All mule deer were outfitted with store-on-board or iridium GPS collars that collected locations every 1–3 hrs (Advanced Telemetry Systems, Isanti, MN, USA; Lotek Wireless, Newmarket, ON, CAN; Telonics, Mesa, AZ, USA). GPS data were organized and managed within R version 4.0.5. Data associated with capture and handling of mule deer were organized and managed within Microsoft Access (Microsoft Office Version Profession Plus 2016).
Data analysis	All movement models and statistical analyses were conducted within R version 4.0.5 with the lme4, stats, suncalc, survival & mgcv packages.

For manuscripts utilizing custom algorithms or software that are central to the research but not yet described in published literature, software must be made available to editors and reviewers. We strongly encourage code deposition in a community repository (e.g. GitHub). See the Nature Portfolio guidelines for submitting code & software for further information.

Data

Policy information about availability of data

All manuscripts must include a data availability statement. This statement should provide the following information, where applicable:
- Accession codes, unique identifiers, or web links for publicly available datasets
- A description of any restrictions on data availability
- For clinical datasets or third party data, please ensure that the statement adheres to our policy

Source Data are provided as a Source Data File with this manuscript. Data underlying this research are available in Dryad (https://doi.org/10.5061/dryad.8kprr4xsj).

Human research participants

Policy information about studies involving human research participants and Sex and Gender in Research.

Reporting on sex and gender	N/A
Population characteristics	N/A
Recruitment	N/A
Ethics oversight	N/A

Note that full information on the approval of the study protocol must also be provided in the manuscript.

Field-specific reporting

Please select the one below that is the best fit for your research. If you are not sure, read the appropriate sections before making your selection.

☐ Life sciences ☐ Behavioural & social sciences ☒ Ecological, evolutionary & environmental sciences

For a reference copy of the document with all sections, see nature.com/documents/nr-reporting-summary-flat.pdf

Ecological, evolutionary & environmental sciences study design

All studies must disclose on these points even when the disclosure is negative.

Study description	We used GPS collar data from 2011–2020 to understand how mule deer (Odocoileus hemionus) may behaviorally compensate en route for phenological mismatches with the green wave.
Research sample	The research sample included adult female mule deer (Odocoileus hemionus; >1-yr-old) that migrated long-distances (134–293 km) from their winter ranges in the Red Desert of south-central Wyoming to their summer ranges in northwestern Wyoming. All deer were captured on their winter ranges via helicopter net-gunning. Capture crews did not capture deer from a single group, and instead, moved throughout the winter range to prevent sampling biases.
Sampling strategy	We did not perform any calculations to determine sample size. In accordance with the minimum sample size of the Central Limit Theorem, we sought to maintain a sample size of at least n = 30 long-distance migrants. Thus, each March and December, we captured new adult female mule deer to replace any deer that died throughout the year or had a failed collar.
Data collection	All mule deer were outfitted with store-on-board or iridium GPS collars that collected locations every 1–3 hrs. During captures, we used an electronic platform scale (± 0.1 kg) to measure body mass (kg) and a portable ultrasound (Ibex, E.I. Medical Imaging, Loveland, CO) to measure maximum rump fat (mm). We used body mass, maximum rump fat, and a body-condition score to estimate percent-scaled ingesta-free body fat (IFBFat). For captures in March, we used an ultrasound to determine pregnancy, including fetal rate (number of fetuses per deer) and fetal development via measures of the fetal eye diameter (mm). To estimate the age of each deer, we extracted the lower right incisiform canine and used a cementum annuli aging technique, which was conducted by the Matson's Laboratory in Manhattan, Montana, USA.
Timing and spatial scale	Our study focused on spring migrations for 2011, 2012, 2014, 2016, 2017, 2018, 2019, and 2020. We only documented the complete spring migration of n = 1 deer in 2013 and n = 1 deer in 2015. We therefore removed these two deer from our analyses. GPS collar data were collected every 1–3 hours. We analyzed how close deer were to peak green-up at 1-km intervals during spring migration. Remotely sensed data included the Normalized Difference Vegetation Index (NDVI) from a time series of MODIS satellite images (MOD09Q1 satellite array; 250-m2 spatial resolution, 8-day temporal resolution). Spatial climate data included mean daily temperature (4-km2 spatial resolution, 1-day temporal resolution) from the Parameter-elevation Regressions on Independent Slopes Model and snow depth (1-km2 spatial resolution, 1-day temporal resolution) from the Snow Data Assimilation System (SNODAS; National Snow and Ice Data Center).
Data exclusions	We were unable to collect complete GPS data from n = 24 animal-years with store-on-board GPS collars either because of collar failures or because we did not recapture the deer and were unable to download GPS data during captures. We removed these animal-years in addition to n = 36 animal-years with incomplete spring migrations because of collar failures or mortalities before or during spring migration. We also removed n = 5 animal-years that spent the winter approximately 70 km north from their historic winter ranges in the Red Desert, n = 10 animal-years that were captured for the first time on their migratory route without prior GPS data, n = 4 animal-years that were recaptured during migration, n = 2 animal-years that returned to their winter ranges in the Red Desert on 17 March and 26 March, and n = 1 outlier that migrated >300 km to a summer range in eastern Idaho. We only documented the complete spring migration of n = 1 deer in 2013 and n = 1 deer in 2015. We therefore removed these two deer from our analyses.<br><br>We removed n = 31 animal-years from the Cox Proportional Hazards (CPH) model because they began spring migration before 20 March (standardized start time). Our final dataset for the CPH model included n = 69 unique mule deer (n = 121 animal-years) and n = 3,645 daily observations.

	We removed n = 25 animal-years with Brownian bridge movement model (BBMM) motion variances greater than 8,000. Our final analyses on stopover use included n = 65 unique mule deer (n = 127 animal-years).
Reproducibility	This study depends on inference from long-term observation and does not use experimental manipulation. Thus, replication from an experimental design was not feasible.
Randomization	All mule deer were captured via helicopter-gunning. The capture crew did not focus on a single group of animals, and instead, moved throughout the study area to prevent sampling biases. Thus, our captures of early, mid, and late migrants are presumed to be random. Using the distribution of the start date of spring migration, we allocated individuals as early, mid, and late migrants. Early migrants started spring migration within the 25% quartile of start dates. Mid-migrants migrants started spring migration between the 25% and 75% quartiles of start dates. Late migrants started spring within the 75% quartile of start dates.
Blinding	Blinding was not relevant to our study because we did not perform any experimental design.

Did the study involve field work? ☒ Yes ☐ No

Field work, collection and transport

Field conditions	Fieldwork (i.e., capture and handling of mule deer) occurred in a semi-arid climate with cold winters (-12.9– -3.9° C, Dec–Feb) and warm summers (2.9–21.6° C, Jun–Aug; 30-yr normal [1991-2020], PRISM Climate Group).
Location	We captured mule deer on their low-elevation winter ranges (2000 m) in the Red Desert of south-central Wyoming, USA (41°47’25” N, 108°57’19” W).
Access & import/export	Mule deer were captured on land owned and managed by the Bureau of Land Management and Sweetwater Royalties. All animal capture and handling protocols were approved by the Wyoming Game and Fish Department (Chapter 33-937) and an Institutional Animal Care and Use Committee at the University of Wyoming (20131111KM00040, 20151204KM00135, 20170215KM00260, 20200302MK00411).
Disturbance	Potential disturbance included the use of a helicopter to capture mule deer. We minimized disturbance by limiting our captures to 5 days each March and December.

Reporting for specific materials, systems and methods

We require information from authors about some types of materials, experimental systems and methods used in many studies. Here, indicate whether each material, system or method listed is relevant to your study. If you are not sure if a list item applies to your research, read the appropriate section before selecting a response.

Materials & experimental systems

n/a	Involved in the study	
☒	☐	Antibodies
☒	☐	Eukaryotic cell lines
☒	☐	Palaeontology and archaeology
☐	☒	Animals and other organisms
☒	☐	Clinical data
☒	☐	Dual use research of concern

Methods

n/a	Involved in the study	
☒	☐	ChIP-seq
☒	☐	Flow cytometry
☒	☐	MRI-based neuroimaging

Animals and other research organisms

Policy information about studies involving animals; ARRIVE guidelines recommended for reporting animal research, and Sex and Gender in Research

Laboratory animals	No laboratory animals were used in this study.
Wild animals	From 2014–2020, we captured n = 220 adult female mule deer (Odocoileus hemionus; >1-yr-old; range = 1–13 years) in the Red Desert near Rock Springs, Wyoming, USA (41° 35’N, 109° 12’W) as part of a long-term study. We recaptured deer each March and December for a total of n = 528 animal-years of data. All deer were captured via helicopter net-gunning. Deer were transported to the processing station via helicopter where researchers deployed GPS collars and collected data on nutritional condition, pregnancy, and age. After data collection (average handling: 18 ± 0.5 min), deer were released from the processing location if captured within 4 km of the processing station; otherwise, deer were ferried back to their capture location via the helicopter. This long-term research project is ongoing (2014–present), and mule deer only exit the study upon mortality from natural or unknown causes or collar failure.

	We also included GPS collar data from a previous study on the Sublette Mule Deer Herd (2011–2013) to analyze movement for n = 27 additional deer (n = 66 animal-years).
Reporting on sex	Our study only included adult female mule deer and did not include any male mule deer.
Field-collected samples	During captures, we extracted the lower right incisiform canine and used a cementum annuli aging technique, which was conducted by the Matson's Laboratory in Manhattan, Montana, USA. All tooth samples were stored in temperatures <0 °C between the time of collection and cementum annuli.
Ethics oversight	All animal capture and handling protocols were approved by the Wyoming Game and Fish Department (Chapter 33-937) and an independent Institutional Animal Care and Use Committee at the University of Wyoming (20131111KM00040, 20151204KM00135, 20170215KM00260, 20200302MK00411).

Note that full information on the approval of the study protocol must also be provided in the manuscript.
